# Kaiso Interacts with p120-Catenin to Regulate β-Catenin Expression at the Transcriptional Level

**DOI:** 10.1371/journal.pone.0087537

**Published:** 2014-02-03

**Authors:** Yang Liu, Qian-Ze Dong, Si Wang, Hong-Tao Xu, Yuan Miao, Liang Wang, En-Hua Wang

**Affiliations:** 1 Department of Pathology, the First Affiliated Hospital and College of Basic Medical Sciences of China Medical University, Shenyang, PR China; 2 Department of Medical Microbiology and Parasitology, College of Basic Medical Sciences of China Medical University, Shenyang, PR China; Rajiv Gandhi Centre for Biotechnology, India

## Abstract

**Background:**

We have reported that p120-catenin could regulate β-catenin transcription in lung cancer cells, but the specific mechanism is unclear.

**Methods and Results:**

In this study, bisulfite sequencing PCR showed that the β-catenin promoter region in SPC-A-1 and LTEP-a-2 lung cancer cell lines has Kaiso binding sites sequences and CpG islands which may combine with Kaiso. The demethylating reagent 5-Aza-2′-deoxycytidine significantly upregulated β-catenin mRNA expression in lung cancer cell lines, whereas expression was significantly reduced following transfection with Kaiso. However, the upregulation of β-catenin mRNA expression after treatment with 5-Aza-2′-deoxycytidine was not reduced by subsequent transfection with Kaiso cDNA. Chromatin immunoprecipitation showed that, in lung cancer cell lines, methylated CpG-dinucleotides sequences combined with Kaiso and the Kaiso binding sites sequence did not. The capacity of Kaiso to combine with p120-catenin isoforms was confirmed by immunoprecipitation.

**Conclusions:**

Based on these results, we concluded that Kaiso participates in the regulation by p120ctn of β-catenin mRNA expression in the lung cancer cell lines.

## Introduction

P120-catenin (p120ctn) and β-catenin are important members of the catenin family, which bind directly with E-cadherin to form the E-cadherin/catenin complex which regulates cell adhesion [1], [2], [3], [4], [5]. Studies have shown that the intracellular domain of E-cadherin is divided into two regions: the juxtamembrane domain (JMD) and the catenin-binding domain (CBD) [6], [7], [8]. p120ctn, a member of the Armadillo gene family, not only combines with the JMD of E-cadherin in the membrane to regulate the E-cadherin-mediated cell-cell adhesion, but also binds with the transcriptional inhibitor Kaiso, to regulate certain transcriptional activities, although the specific mechanism remains controversial [5]. β-catenin binds to the CBD of E-cadherin, to mediate E-cadherin binding with α-catenin and the actin cytoskeleton, which regulates cell adhesion [1], [9], [10].

Human p120ctn isoforms 1 to 4 differ according to the start codon from which translation is initiated. Additional isoforms are derived from the variable inclusion of exons A, B, and C [11], [12], [13]. Although the different isoforms have divergent N- and C-terminal ends, they share the central Armadillo repeat domain, which is used to bind E-cadherin, and thus to regulate cell-to-cell adhesion. Isoforms that arise from alternative splicing events may have tissue- or cell-specific functions [14], [15], [16].

In our previous study of p120ctn in lung cancer, we not only found expression of p120ctn and its isoforms in lung cancer cells but also, unexpectedly, found that p120ctn regulated β-catenin mRNA expression [17], [18], [19], [20]. To the best of our knowledge, this was the first report describing the regulation of β-catenin transcription by p120ctn. However, it is unclear whether p120ctn is involved in translational regulation of β-catenin.

Kaiso is a member of the broad complex, Tramtrak, Bric à brac/Pox virus and zinc finger (BTB/POZ) subfamily of zinc finger proteins, that was originally identified in a yeast-two-hybrid screen for binding partners of p120ctn [5], [21], [22]. However, unlike any of the previously characterized POZ proteins, Kaiso exhibits dual-specificity DNA binding: KBS (Kaiso binding sites), which recognize a sequence-specific DNA consensus, TCCTGCnA, as well as methylated CpG-dinucleotides [21], [22], [23]. Our previous study demonstrated that Kaiso could bind to p120ctn in lung cancer cells [24]. Although known to be a component of the Kaiso/p120ctn complex, each individual p120ctn isoform might possess a different affinity for Kaiso [5], [25], [26]. At the same time, we also found that the promoter region of β-catenin is methylated in lung cancer. Thus, in this study we aimed to investigate the hypothesis that the β-catenin promoter region contains a methylated CpG dinucleotide sequence that is recognized and bound by Kaiso to mediate indirect regulation of β-catenin transcription.

## Materials and Methods

### 1. Cell Culture

Human lung cancer cell lines A549, NCI-H460 (H460), SPC-A-1(SPC) and LTEP-a-2(LTE) were cultured in either DMEM or RPMI 1640 medium (both from Invitrogen, Carlsbad, CA, USA) supplemented with 10% fetal calf serum (FCS; Invitrogen), 100 IU/ml penicillin (Sigma, St. Louis, MO, USA), and 100 µg/ml streptomycin (Sigma). Cells were grown on sterile culture dishes and passaged every 2 days, using 0.25% trypsin (Invitrogen).

The A549 and H460 cell lines were obtained from the American Type Culture Collection (Manassas, VA, USA). SPC and LTE cell lines were obtained from Shanghai Cell Bank (Shanghai, China).

### 2. Treatment with 5-Aza-2′-deoxycytidine (5-Aza-CdR)

Cultivated lung cancer cells were treated either with 5-Aza-2′-deoxycytidine (5-Aza-CdR) (Sigma) at different concentrations dissolved in culture medium. methylation specific PCR (MSP) and MTT assays were used to select the 5-Aza-CdR concentration which enables β-catenin promoter CpG island demethylation without significant effects on cell growth (7 µmol/L) which was then used for subsequent demethylation processing and cells were collected after continued culture for 48 h.

### 3. cDNA Plasmids and Transfection

p120ctn-1A and p120ctn-3A cDNA plasmids and Kaiso cDNA plasmid (gifts from Dr. Reynolds, Vanderbilt University, Nashville, USA), and p120ctn-1A and p120ctn-3A cDNA plasmids with C-terminal MYC and DDK Tagged in pCMV6-Entry (PS100001, Origene) were transfected using Lipofectamine 2000 (Invitrogen) into cells, following the manufacturer’s instructions. The empty plasmid was used as a negative control. Protein expression by the transfected cells was confirmed by Western blot analysis.

### 4. Reverse Transcription-polymerase Chain Reaction (RT-PCR)

Total RNA was extracted from cells with TRIzol Reagent (Invitrogen). RT-PCR was performed with the RNA PCR Kit (AMV) Version 3.0 (TaKaRa Bio Inc., Dalian, Liaoning, China), according to the manufacturer’s instructions. The primer sequences were as follows: β-catenin: 5′-CATCCGGAAGAAACTGGT-3′; 5′-TCCCACA AAGCCAACTC-3′ GAPDH: 5′-TACTAGCGGTTTTACGGGCG-3′; 5′-TCG AACAGGAGGAGCAGAGAGCGA-3′ After electrophoresis on a 1.5% agarose gel, the PCR product bands were visualized with BioImaging Systems and quantitated with Labworks Image Acquisition and Analysis Software (UVP Inc., Upland, CA, USA). The mRNA levels were normalized to the amount of GAPDH mRNA.

### 5. Western Blot Analysis

Cells were lysed in Radio Immune Precipitation Assay (RIPA) (p0013, Beyotime, Shanghai, China) with 1 mM phenylmethylsulfonyl fluoride (PMSF, Sigma). The protein concentration was determined using Coomassie brilliant blue (Sigma), with bovine serum albumin (BSA) (Invitrogen) as the standard. Each sample (50 µg) was separated by 8% or 12% SDS-PAGE for 60 min and transferred (100 V or 50 V, 2 h) to a polyvinylidene fluoride (PVDF) membrane (Millipore, Billerica, MA, USA). After blocking with 1% BSA in Tris-buffered saline-Tween (TBST; 20 mM Tris-HCl, 500 mM NaCl, 0.05% Tween-20), the membrane was incubated overnight at 4°C with a mouse monoclonal antibody against either p120ctn (1∶400; BD Transduction Laboratories, Franklin Lakes, NJ, USA), Kaiso (H-154) (1∶400, Sc-98589, Santa Cruz Biotechnology, Santa Cruz, CA, USA), anti-Kaiso antibody [6F/6F8] (Ab12723, Abcam, Cambridge, MA, USA) or myc (1∶800, Beyotime, Shanghai, China). After incubation with peroxidase-coupled anti-mouse-IgG (SABC, Beijing, China) at 37°C for 2 h, the protein bands were visualized using ECL (Pierce, Rockford, IL, USA) and detected using the BioImaging Systems (UVP Inc.). The relative protein levels were calculated by comparison to the amount of GAPDH protein.

### 6. Quantitative Real-time PCR (qRT-PCR)

Total RNA was extracted from cultivated lung cancer cells using a Total RNA Isolation Classic Kit (TIANGEN, Beijing, China). For quantitative RT-PCR (qRT-PCR), first-strand cDNA was synthesized from total RNA using the TIANScript RT Kit (TIANScript cDNA First-Strand Synthesis System Kit; TIANGEN) according to the manufacturer’s instructions. The resulting cDNA was used as a template for qRT-PCR using an ABI 7900 sequence detector (Applied Biosystems). The relative levels of gene expression were calculated by the 2-^△△Ct^ method. Experiments were repeated in triplicate. GAPDH was used as the reference gene, the sequences of the primer pairs were as described in Section 2.4.

### 7. β-catenin Promoter Assay

Methyl Primer Express (v1.0) was used to analyze the CTNNB1 gene promoter region (−1,124–11,114 bp).

### 8. Bisulfite Sequencing PCR (BSP) of β-catenin Promoters

DNA from lung cancer cells was extracted using a cell/tissue genomic DNA extraction kit (DP3402, BioTeke Corporation, Beijing, China) and then treated with sodium bisulfite using the EZ DNA Methylation-Gold Kit (D5005, Zymo Research, Orange County, CA, USA) according to the manufacturer’s instructions. The β-catenin promoter-specific primers for bisulfite sequencing were constructed using promoter sequence data and primer design software. Primer sequences are shown in Table 1. The primers were designed to amplify a CpG-rich region of the promoter spanning 189 CpG sites (19 CpGCpG sites). The PCR products were purified using the multifunctional DNA purification extraction kit (DP1501, BioTeke Corporation) and ligated into the pUM-T simple vector (DP6803, BioTeke Corporation). At least ten separate clones each were chosen for sequence analysis by BiQ Analyzer.

**Table 1:** β-catenin promoter-specific primers for bisulfite (BSP) sequencing.

Name	Sequence (5′-3′)	Length	Tm	size
1 F	TGCGATTTAGGTTTAGTAGGGAGTGT	26	62.4	345
1 R	AATATCCTCCCCTATCCCAAACC	24	62	
2 F	ATAGGGGAGGATATTAGGGTTATT	24	56.7	336
2 R	TAACGCCGCACAAAAAACTCTTAT	24	62.7	
3-1 F	GGAGGAAGGTTTGAGGAGTAGTTTTAG	27	62.3	387
3-1 R	CCGCCTACCATCCG/AACTCCTATA	24	65.7	
3-2 F	TATAGGAGTTCGGATGGTAGG	21	54	232
3-2 R	CCCCAAAACTAATAAAACTTAAAATAAC	28	58.8	
3-3F	CGGCGTTATTTTAAGTTTTTCG	22	59.5	104
3-3 R	AAACTACTCCTCAAACCTTCCT	22	53.9	
4F	GTTGAAAAATTAAGATATGGGTTAG	25	54.7	355
4R	CTATAAACCTAAACTAATATATTCATATC	29	51.4	

### 9. Chromatin Immunoprecipitation (ChIP)

Chromatin immunoprecipitation (ChIP) was performed using a ChIP kit (Millipore, Billerica, MA, USA) according to the manufacturer’s instructions with some modifications. Briefly, 4×10^7^ cells were cross-linked by adding formaldehyde to the culture medium at a final concentration of 1% (v/v). After fixation at room temperature for 10 minutes, L-glycine (final concentration, 0.125 mol/L) was added to terminate the cross-linking reaction. Then cells were washed twice with cold PBS containing Protease Inhibitor Cocktail II, and collected by centrifugation at 720 ×*g* for 10 minutes at 4°C. Chromatin of fixed cells was solubilized by EZ-Zyme™ Chromatin Prep Kit (Millipore), and the lysates were subjected to electrophoresis on a 2% agarose gel to verify the DNA fragments with an average size of 200 bp to 500 bp in length. The chromatin solution was subjected to EZ-ChIP™ Chromatin Immunoprecipitation (Millipore). The chromatin solution was precleaned by incubation with Protein G agarose for 1 h at 4°C and then incubated with anti-Kaiso antibody - ChIP Grade (Abcam), normal mouse IgG (Santa Cruz Biotechnology), or anti-RNA Polymerase II (Santa Cruz Biotechnology). Antibody complexes were precipitated with Protein G agarose, washed with ChIP wash buffer, and eluted twice with 200 µl elution buffer. Cross-linking was reversed by adding 5 M NaCl and incubating at 65°C overnight. DNA was purified by incubation with 0.5 M EDTA, 1 M Tris-HCl, and proteinase K at 45°C for 2 h and then subjected to PCR amplification using a primer set designed to amplify β-catenin promoter sequences containing the CpG sites and the KBS element.

### 10. Luciferase Reporter Assay

The human CTNNB1 gene promoter (positions-614 to -270, which contains the methylation sites in the promoter region) and the KBS sequence (5′-GAAATTAAATCTCCTGCAATAGACTATA-3′) were amplified from genomic DNA by PCR, inserted between the restriction enzyme sites XhoI and KpnI of the Firefly luciferase reporter vector pGL3-Basic (E1751, Promega, CA, USA), and validated by sequencing. The construct with a mutation of the KBS sequence (5′-GCGCGCCGAGTCATGCAGCTGCTCTCC-3′) was generated using mutagenic oligonucleotide primers, according to the manual of the GeneTailor Site-Directed Mutagenesis System (Invitrogen). All the reporter plasmids were purchased from Biotime Biotechnology (Beijing, China).

Lung cancer cells were plated in a 12-well plate, and co-transfected with luciferase reporter vectors and Renilla luciferase reporter pRL-TK Vector (E2241, Promega) into the cell lines with Kaiso-pcDNA3 vector or control vector (empty-pcDNA3 vector) using Lipofectamine 2000 (Invitrogen). Forty-eight hours after transfection, the relative luciferase activity, expressed as the ratio of Firefly to Renilla, was measured by Dual-Luciferase Reporter Assay System (E1910, Promega), according to the manufacturer’s instructions.

### 11. Immunoprecipitation

Cells were lysed in precooled IP cell lysis buffer (Beyotime, Shanghai, China) with 1 mM PMSF (Sigma) on ice. The lysate was centrifuged at 12,000 rpm for 10 min at 4°C and the supernatant was quantified by BSA, and equal amounts of total protein were used for immunoprecipitation with the anti-Kaiso (Abcam), mouse IgG (A7028, Beyotime), and PBS. Following the addition of ProteinA+G agarose (P2012, Beyotime), and slowly shaking for 3 h at 4°C, the immunocomplexes were centrifuged at 2,500 rpm for 5 min, washed with PBS and subjected to SDS-PAGE.

### 12. Statistical Analysis

All statistical analyses were performed using SPSS 11.5 for Windows software. Data from cells in different experimental groups were compared using the independent samples t-test or ANOVA. *P* values <0.05 were considered statistically significant.

## Results

### 1. The β-catenin Promoter has KBS Sequences and CpG Islands

**Figure 1:**
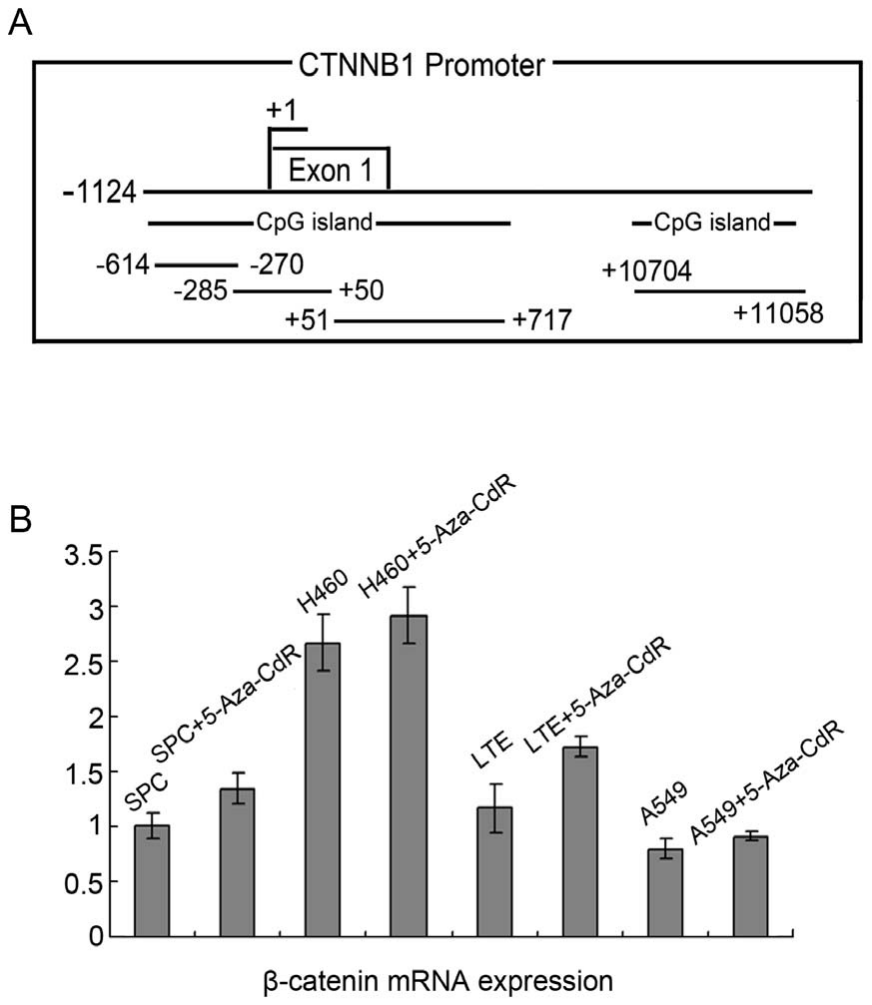
Figure 1. β-catenin mRNA expression was upregulated in lung cancer cell lines following treatment with 5-Aza-CdR. A: −1,124–11,114 bp revealed the presence of two CpG islands (positions −1,124–876 and 10,676–11,114). B: Treatment of lung cancer cell lines with 5-Aza-CdR resulted in varying levels of increased β-catenin mRNA expression. Statistical analysis by t-test showed increased β-catenin mRNA expression in lung cancer cell lines, which were treated with 5-Aza-CdR, compared to untreated cells (SPC, *P* = 0.030; H460, *P* = 0.308; LTE, *P = *0.035; A549, *P = *0.151).

**Figure 2:**
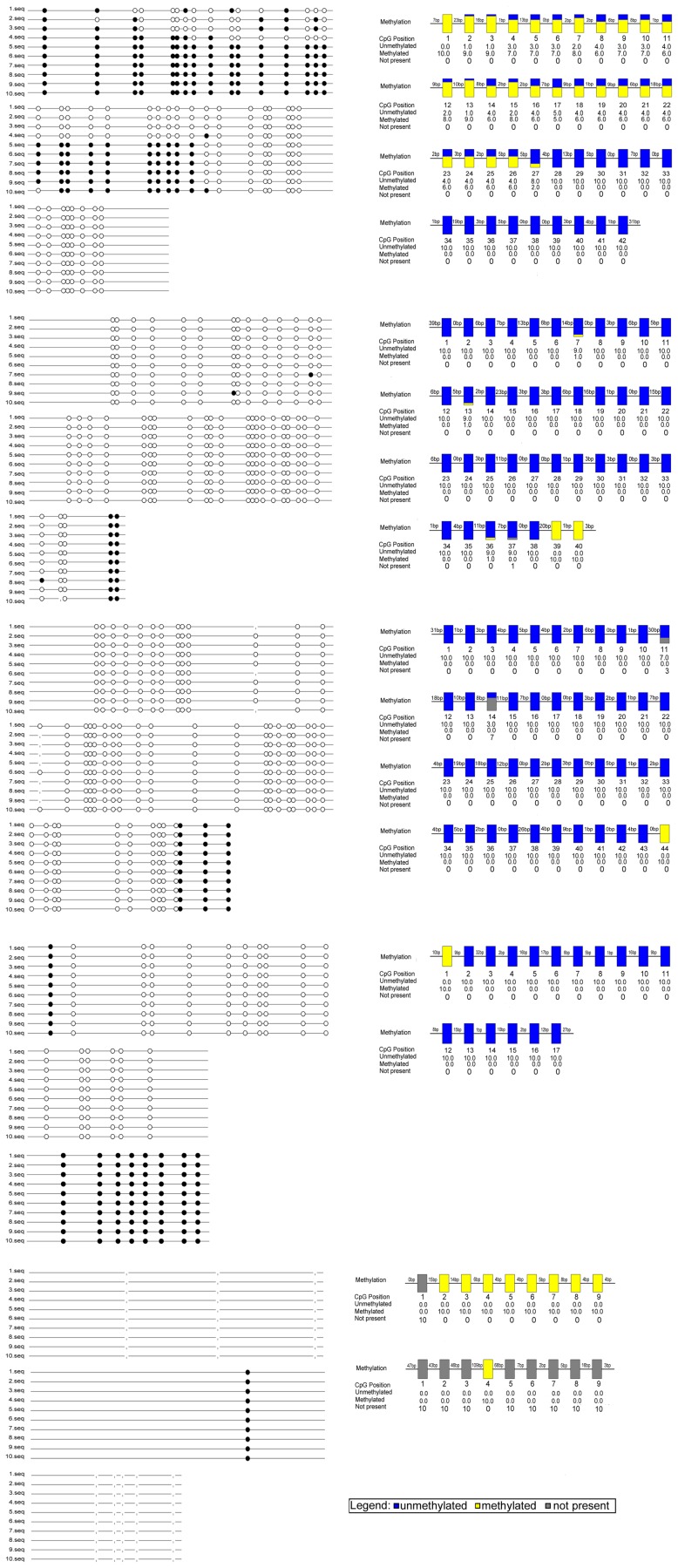
Methylation status of the β-catenin promoter region in LTE. Mapping of the BSP results of lung cancer cell lines LTE, showing the methylation status of the β-catenin promoter region. The filled circles represent methylated CG sites, hollow circles represent unmethylated CG sites. Yellow indicates methylation, blue indicates unmethylated, gray indicates no CG site.

**Figure 3:**
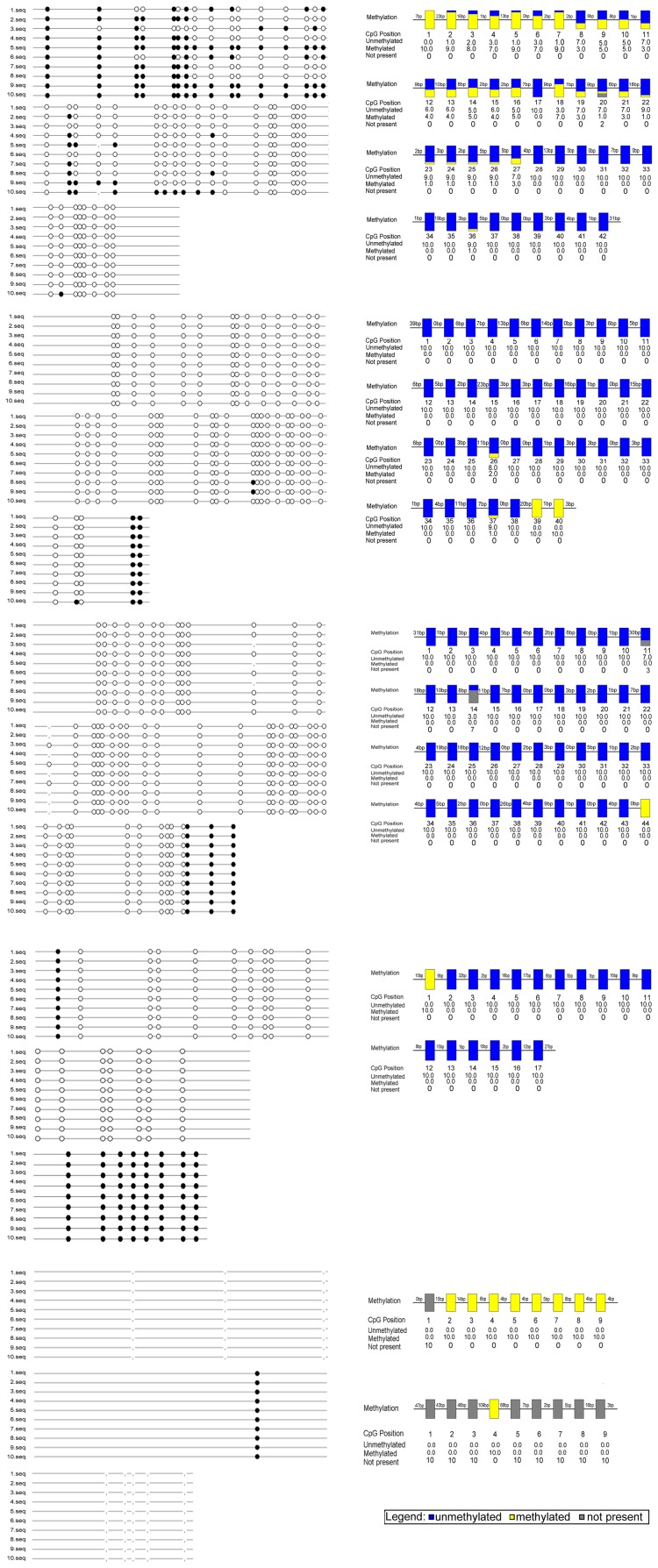
Methylation status of the β-catenin promoter region in SPC. Mapping of the BSP results of lung cancer cell lines SPC, showing the methylation status of the β-catenin promoter region. The filled circles represent methylated CG sites, hollow circles represent unmethylated CG sites. Yellow indicates methylation, blue indicates unmethylated, gray indicates no CG site.

Analysis of the CTNNB1 gene promoter region (−1,124–11,114 bp) revealed the presence of two CpG islands (Fig. 1A), and treatment of lung cancer cell lines with 5-Aza-CdR (7 µmol/L) for 48 h resulted in varying levels of increased β-catenin mRNA expression (Fig. 1B). According to the results, we choose human lung cancer cell lines LTEP-a-2(LTE) (Fig. 2) and SPC-A-1(SPC) (Fig. 3), in which the increase were more significant, to further study, and using Methyl Primer Express (v1.0) revealed the presence of two CpG islands (positions −1,124–876 and 10,676–11,114) which contained 189 single CG sites, including 19 CG-dinucleotides. Unexpectedly, we also identified the KBS sequence (TCCTGCnA) in the CTNNB1 gene promoter region, which included the TCCTGCAA sequence at position 2,684 bp (data not shown).

### 2. High Expression of Kaiso Suppresses β-catenin mRNA Expression in Lung Cancer Cell Lines that are not Treated with 5-Aza-CdR

**Figure 4:**
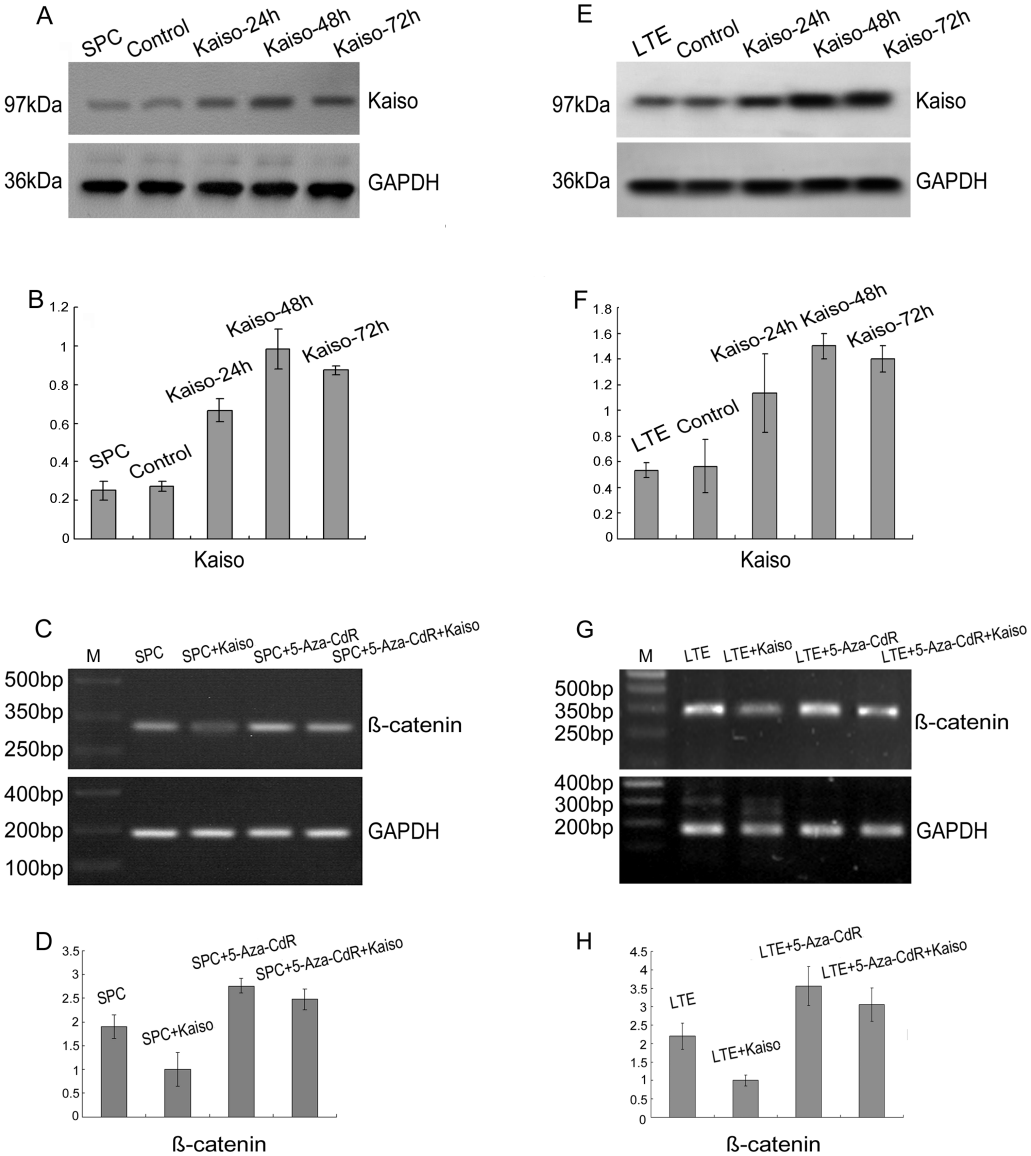
Kaiso suppresses β-catenin mRNA expression in cell lines that have not been treated with 5-Aza-CdR. Lung cancer cell lines SPC (A) and LTE (E) were transfected with Kaiso cDNA plasmid. The effect of transfection at different time points was identified by Western blot. The Kaiso-specific band appears at 97 kDa. Statistical analysis of SPC (B) or LTE (F) by t-test showed a significant increase Kaiso expression in the cells transfected with Kaiso cDNA plasmid compared with the control cells (B: *P* = 0.003 for 24 h, *P* = 0.005 for 48 h, and *P*<0.001 for 96 h, respectively; F: *P* = 0.065 for 24 h, *P* = 0.007 for 48 h; *P* = 0.009 for 96 h, respectively). Analysis of β-catenin mRNA expression in SPC following treatment of using RT-PCR (C) and Real-Time PCR by ANOVA (D) showed that treatment with 5-Aza-CdR demethylation reagent (7 µmol/L) for 48 h resulted in significant upregulation (*P* = 0.007), whereas high Kaiso expression significantly down-regulated β-catenin mRNA expression (*P* = 0.004). The expression of β-catenin mRNA expression in cells treated with 5-Aza-CdR reagent did not change significantly in the presence of high Kaiso expression (*P* = 0.062). Similar effects were observed in the LTE cell line (G and H; H: *P* = 0.003, *P* = 0.001, and *P* = 0.055 accordingly).

Each lung cancer cell line was transfected with the Kaiso cDNA plasmid and Kaiso protein expression confirmed by Western blot analysis at different time points (Fig. 4A and B for SPC; Fig. 4E and F for LTE). According the transfection effect, lung cancer cell lines at 48 h after transfection were selected and the effect of Kaiso on β-catenin mRNA expression was analyzed by PCR (Fig. 4C and D for SPC; Fig. 4G and H for LTE). We found that the high expression of Kaiso suppressed β-catenin mRNA expression in each lung cancer cell line. However, in cell lines treated with 5-Aza-CdR prior to transfection with Kaiso cDNA plasmid, β-catenin mRNA expression was still increased.

### 3. The Kaiso Binding Site of the β-catenin Promoter Region Contains Methylated CpG Dinucleotide Sequences, Rather than the KBS Sequence

**Figure 5:**
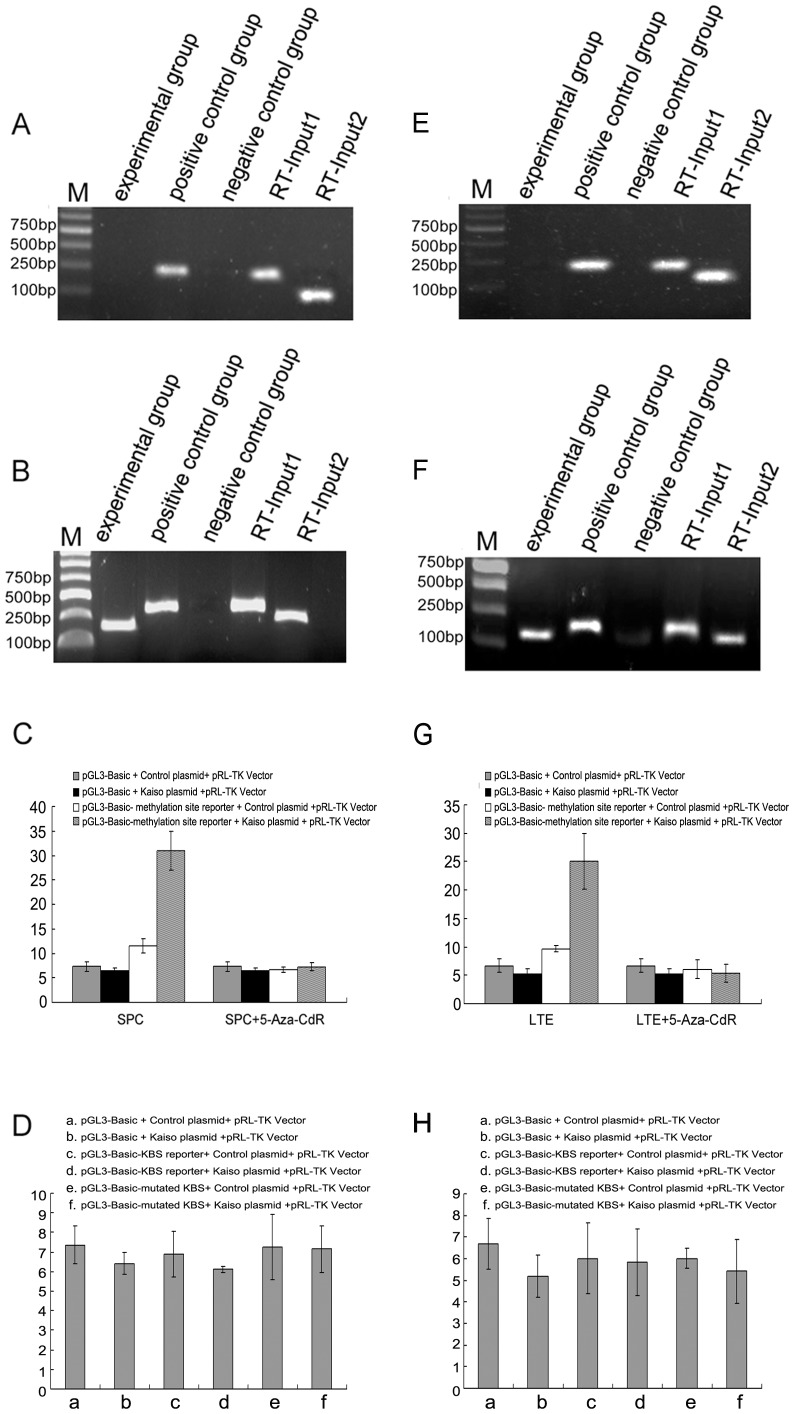
Kaiso binds the β-catenin promoter region via methylated CpG dinucleotide sequences. No specific bands appear in the KBS binding region (A), while a specific band appears in the methylated CpG dinucleotide sequence region in SPC cell line (B). C: Luciferase reporter vectors and pRL-TK Vector were co-transfected into SPC cells with either the control vector or kaiso expressing plasmid DNA. They were then compared with cells treated with demethylating agents to assess the importance of the Kaiso binding domain. Statistical analysis by ANOVA indicated that the relative luciferase activity in the cell group with methylation site reporter vectors and Kaiso plasmid were higher than the other cell groups (*P* = 0.000, F = 83.018). No apparent changes in activity were observed in the SPC cells that were treated with demethylating agents (*P* = 0.374, F = 1.187). D: Luciferase activity obtained from the mutant construct showed no difference in any of these conditions (*P* = 0.674, F = 0.641). Similar results of ChIP (E, F) and luciferase analyses (G: *P* = 0.000, F = 37.703; *P* = 0.569, F = 0.718; H: *P* = 0.762, F = 0.513, respectively) were observed in the LTE cell line.

Based on our experimental results, we found that the regulatory effects of Kaiso on β-catenin transcription are related to methylation, and we observed that the β-catenin promoter region contains methylated CpG dinucleotide sequences. Therefore, we investigated the presence of methylated CpG dinucleotide sequences within the β-catenin promoter region and the effects on Kaiso binding in lung cancer cell lines. ChIP studies showed that Kaiso binds with the β-catenin promoter region through methylated CpG dinucleotide sequences. We also identified the KBS sequence within the β-catenin promoter region, although specific binding of this sequence with Kaiso was not detected by ChIP. Therefore, we speculate that Kaiso binds to the β-catenin promoter region via methylated CpG dinucleotide sequences, rather than the KBS sequence (Fig. 5A and B for SPC; Fig. 5E and F for LTE). In addition, we used luciferase reporter assays to confirm the hypothesis. Dual-luciferase assay indicated that the methylation site was essential for the CTNNB1 promoter activities; however, the results using the mutated KBS sequence in the promoter showed that the KBS sequence was not required.

### 4. p120ctn Isoforms 1A and 3A Exhibit Different Binding Capacity with Kaiso

**Figure 6:**
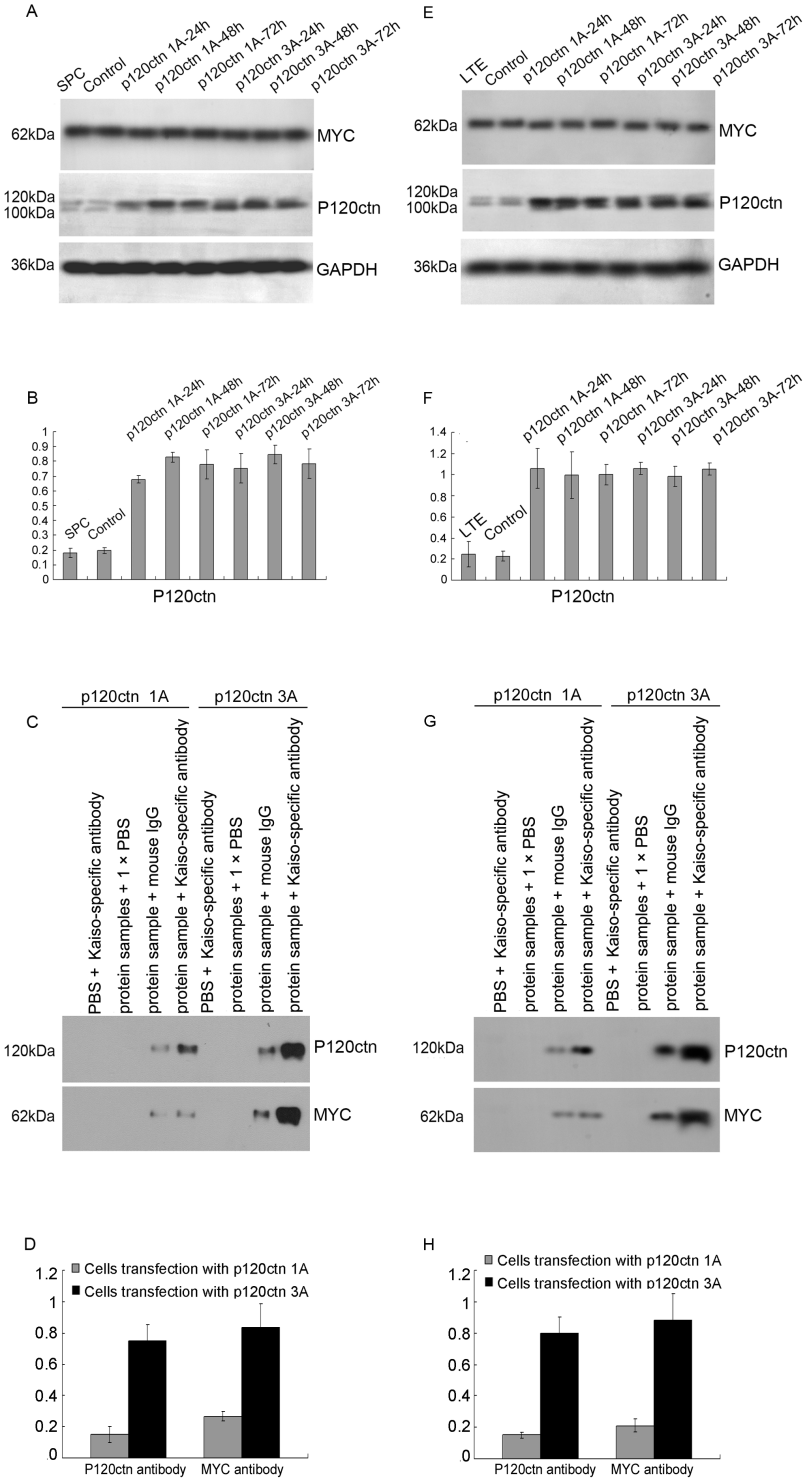
The binding of p120ctn isoforms 1A and 3A with Kaiso. We introduced plasmids encoding DDK-MYC tagged p120ctn isoforms 1A and 3A cDNA into the lung cancer cell lines, and verified the effect of transfection by Western blot using p120ctn and MYC specific antibodies. P120ctn specific bands were detected at 120 kDa and 100 kDa and MYC specific bands were detected at 62 kDa in following transfection of the SPC (A) and LTE (E) cell lines. Statistical analysis by t-test in SPC (B) showed that cells transfection with p120ctn-1A (*P*<0.001 for 24 h, *P*<0.001 for 48 h, and *P* = 0.007 for 72 h, respectively) and -3A (*P* = 0.008 for 24 h, *P* = 0.001 for 48 h, and *P* = 0.008 for 72 h, respectively) showed significant expression, compared with control cells. Similar results to those in the SPC cell line were seen in the LTE line (F: in the cells transfection with p120ctn-1A, *P* = 0.013 for 24 h, *P* = 0.023 for 48 h, and *P* = 0.001 for 72 h, respectively; in the cells transfection with p120ctn-3A, *P*<0.001 for 24 h, *P* = 0.002 for 48 h, and *P*<0.001 for 72 h, respectively). Co-immunoprecipitation results confirmed that Kaiso formed complexes with proteins expressed by p120ctn isoform plasmid transfected SPC (C) and LTE (G) cell lines, and the statistical analysis by t-test in SPC (D) and LTE (H) showed that the binding ability of kaiso with p120ctn isoform 1A was significantly less than that of p120ctn isoform 3A (D: *P*<0.001 for p120ctn, *P* = 0.002 for MYC, respectively; H: *P*<0.001 for p120ctn, *P*<0.001 for MYC, respectively).

Kaiso, as a binding partner of p120ctn, exhibit dual-specificity DNA binding: Kaiso recognizes a sequence-specific DNA consensus, TCCTGCnA, as well as methylated CpG-dinucleotides, in addition to its ability to combine with p120ctn. In the present study, we introduced p120ctn isoforms 1A and 3A into lung cancer cell lines by transfection with cDNA plasmids containing a DKK-MYC tag and the effects of transfection were confirmed by Western blot analysis (Fig. 6A and B for SPC; Fig. 6E and F for LTE). Investigation of the p120ctn isoforms with Kaiso by immunoprecipitation showed that p120ctn isoforms 1A and 3A in lung cancer cell lines are able to bind to Kaiso, although the binding capacity of isoform 1A was lower than that of isoform 3A (Fig. 6C and D for SPC; Fig. 6G and H for LTE).

## Discussion

We have previously reported that decreased expression of p120ctn down-regulates β-catenin mRNA expression in the lung cancer cell lines LTEP-a-2(LTE) and SPC-A-1(SPC) [19]. However, the specific regulation mechanism is unclear and this, therefore, formed the focus of the present study.

Cancers often exhibit aberrant methylation of gene promoter regions, which is linked to abnormal gene transcription [27], [28], [29], [30]. We have also detected that the β-catenin promoter region is methylated in lung cancer by methylation specific PCR (MSP). In addition, real-time PCR analysis showed that β-catenin mRNA expression was upregulated in lung cancer cell lines following treatment with 5-Aza-CdR. As a result, we postulated that the β-catenin promoter region has methylated CpG islands and that methylation in the β-catenin promoter region is implicated as a major factor influencing the regulation of β-catenin mRNA expression in lung cancer.

However, the proportion and location of CpG islands and specifically, the presence of CpG dinucleotide sequences in the β-catenin promoter region remain to be established. Therefore, we used Methyl Primer Express v1.0 to analyze the CTNNB1 gene promoter region (−1,124–11,114 bp). We identified two CpG islands in the promoter region, containing 189 single CG sites, including 19 CG-dinucleotides. Among the 19 CG-dinucleotides, only one CG-dinucleotide was shown to be methylated by BSP sequencing. We also unexpectedly identified the KBS sequence in the CTNNB1 gene promoter region.

Kaiso, which is an important member of the BTB-POZ protein family, has been confirmed with transcriptional repressor function, such as its ability to directly repress canonical Wnt gene targets (Siamois, c-Fos, Cyclin-D1, and c-Myc) [31], then whether kaiso involved in β-catenin mRNA expression itself? To investigate this issue, we introduced a Kaiso cDNA plasmid into lung cancer cell lines. Following confirmation of recombinant protein expression by Western blotting, we showed that high expression of Kaiso significantly inhibited the transcription of β-catenin. However, Kaiso was not able to mediate this inhibition following treatment with the 5-Aza-CdR demethylating agent. These results indicate that the regulatory effects of Kaiso on β-catenin mRNA expression are influenced by methylation.

Based on these results, we investigated whether the transcriptional repressor function of Kaiso depends on its binding with the KBS sequence or the methylated CpG dinucleotide sequence within the β-catenin promoter region in lung cancer cells. ChIP and Luciferase analysis confirmed that Kaiso combines with the β-catenin promoter region via the CpG dinucleotide sequence rather than the KBS sequence.

It is known that Kaiso combines with p120ctn in addition to recognizing and binding specific DNA sequences within the promoter region. Our previous study demonstrated that Kaiso binds to p120ctn in lung cancer cells, and that p120ctn 3 is likely to be the major isoform [24], [25], [26]. However, we did not confirm that p120ctn isoform 1A combines with Kaiso. This issue was further investigated in the present study by introduction of plasmids encoding DDK-MYC-tagged p120ctn isoforms 1A and 3A cDNA into lung cancer cell lines. Co-immunoprecipitation confirmed that p120ctn isoform 1A also combined with Kaiso, although its binding ability was significantly less than that of p120ctn isoform 3A.

We then speculated that the mechanism by which decreased expression of p120ctn down-regulated β-catenin mRNA expression in the lung cancer cell lines SPC and LTE is based on the decreased binding of p120ctn with Kaiso. This results in increased combination of Kaiso with the methylated CpG dinucleotide sequence within the β-catenin promoter region. This would result in increased binding of the β-catenin promoter region with Kaiso and thus increased transcriptional inhibition of β-catenin.

However, it is interesting that we also found that p120ctn isoform 1A noticeably increased the expression of β-catenin mRNA in the lung cancer cell lines A549 and NCI-H460 (H460) [18], although the methylation of the β-catenin promoter region was not detected in the cells. Therefore, it can be speculated that p120ctn regulates β-catenin mRNA expression by an alternative mechanism in these cells. Many questions regarding the regulation of β-catenin mRNA expression have yet to be investigated. Although it is clear that Kaiso participates in the regulation by p120ctn of β-catenin mRNA expression in the lung cancer cell lines, identification of the region of the β-catenin promoter that is methylated remains to be elucidated.
